# Health-care access and utilization among individuals with low back pain in Iran: a WHO-ILAR COPCORD study

**DOI:** 10.1186/s12913-020-05727-z

**Published:** 2020-09-17

**Authors:** Seyedeh Tahereh Faezi, Azarakhsh Baghdadi, Mohammad Nejadhosseinian, Maziar Moradi-Lakeh, Mir Saeed Yekaninejad, Kourosh Holakoui, Nasrin Moghimi, Mahnaz Sandoughi, Ali Dehghan, Arash Tehrani Banihashemi, Maryam Ghadimi, Fereydoun Davatchi

**Affiliations:** 1 Rheumatology Research center, Tehran University of Medical Sciences, North Amirabad street, Tehran, 1411713137 Iran; 2 Russell H. Morgan Department of Radiology and Radiological Sciences, Johns Hopkins University School of Medicine, Baltimore, MD USA; 3 Preventive Medicine and Public Health Research Center, Iran University of Medical Sciences, Tehran, Iran; 4 Department of Epidemiology and Biostatistics, School of Public Health, Tehran University of Medical Sciences, Tehran, Iran; 5 Department of Internal Medicine, Faculty of Medicine, Kurdistan University of Medical Sciences, Sanandaj, Iran; 6 Department of internal medicin Ali Ebn Abitaleb Hospital, Zahedan university of medical sciences, Zahedan, Iran; 7 Department of Internal Medicine, Shahid Sadoughi Hospital, Shahid Sadoughi University of Medical Sciences, Yazd, Iran; 8 Preventive Medicine and Public Health Research Center, Community Medicine Department, Iran University of Medical Sciences, Tehran, Iran

**Keywords:** Low back pain, Rheumatologic diseases, Health-care access, COPCORD, Epidemiology, Public health

## Abstract

**Background:**

Low back pain (LBP) is a major contributor to chronic pain and disability. The purpose of this study was to evaluate health-care access and utilization among patients with LBP in Iran. We also sought to study the pattern and characteristics of care-utilization behavior in these patients.

**Methods:**

Data from the Community Oriented Program for Control of Rheumatic Diseases (COPCORD) were used for this study. Three cities (Zahedan, Sanandaj, Yazd) were selected to represent the Iranian population, with different socioeconomic status and ethnic, cultural, and religious background. Demographic data, acute or chronic LBP, disability index, and utilizing care from conventional medicine (CM), allied health providers (AHP), and complementary and alternative medicine (CAM) providers were recorded.

**Results:**

Of 9101 patients, 38.6% reported LBP. Only 3.3% did not utilize care of any kind, 66.7% referred to CM providers, 20.8% to AHP, and 9.2% to CAM care. Health-care utilization was higher in female patients, older age, higher education, and higher disability index.

**Conclusions:**

The findings of this study indicate a high rate of health-care utilization among patients with LBP in Iran. CM is the most prevalent health-care resource sought by patients. These findings could be used as a framework in developing more efficient health-care programs according to the needs of specific populations.

## Background

Low back pain (LBP) is one of the most common symptoms experienced by people of all ages globally [1]. A specific identifiable neurological disorder (e.g., myelopathy) is found only in 10 % of individuals, with the vast majority of patients suffering from non-specific low back pain [2]. The results of the Global Burden of Disease Study 2017 indicate that LBP is the leading cause of years lived with disability in 126 of the 195 countries that study. Also, nearly 540 million people are affected by LBP at any point in time. Further, LBP caused 65 million life-years lost in the same year, with an increase of 30% from 1990 [3]. In 2015, LBP and neck pain were the fourth leading cause of disability-adjusted life years (DALYs) globally [4]. Low- and middle-income African, Asian, and Middle-eastern countries have suffered the largest increase in disability caused by LBP. While data on the financial burden of LBP is scarce from the developing world, in the United States alone, the direct and indirect costs of LBP is estimated to be at least $84.1 billion a year [5]. Moreover, people who retire early due to chronic LBP have about 87% less total wealth and income-producing assets, which combined with the lack of an effective social support and an all-inclusive health-care system in most of the developing world, is indicative of a disaster in the making for low- to middle-income countries [2].

While the high prevalence and costs of LBP have encouraged clinicians of many medical disciplines to develop and study the disease, an ideal prevention or treatment protocol is as elusive as ever. A variety of non-pharmacologic and pharmacologic therapies have been studied by researchers around the world, with varying results [2]. This has all but added to the confusion for the clinicians and patients alike. The disciplines involved in the treatment of LBP can be loosely gathered in three categories: conventional medicine (CM), which includes physicians of any specialty, allied health professionals (AHP), including physical therapists and chiropractors, and complementary and alternative medicine (CAM), including homeopathists, bone-setters, and herbal medicine providers. Although the first two groups are responsible for the majority of visits, the renewed interest for CAM as a global phenomenon has shifted many patients, especially those with chronic conditions, away from conventional medical treatments [6]. The high cost and limited access to proper health care, together with cultural issues, may play a role in this trend. Therefore, in order to overcome the health-care challenges of musculoskeletal disabilities, especially low back pain, a public health program to provide a model and structure for proper management is needed. The first step towards this goal would be to study the epidemiology of LBP, along with access and the desire to utilize public health-care services [7].

The Community Oriented Program for Control of Rheumatic Diseases, or COPCORD, was an initiative started in 1983 by the world health organization (WHO) and international league of associations for rheumatology (ILAR) with the same concepts in mind. As a ‘community program for control of rheumatic diseases’, this program was created for recognition, prevention, and control of rheumatic disorders in the developing countries. So far, 21 countries have participated in COPCORD, and multiple publications have elucidated different aspects of rheumatologic disorders in the developing world [8].

The results of the COPCORD initiative have shaped our understanding of the global epidemiology of LBP in the developing world. A recent study combining the Latin-American COPCORD data shows that LBP affects 6.59% of the population, with a high impact on the quality of life of young adults. Also, LBP has a significant association with social variables including gender inequality index, human development index, and income inequality [9]. Further, the Mexican and Portuguese COPCORD data reveals that being overweight/obese, female gender, medical and musculoskeletal comorbidities, and age affect the prevalence of LBP and its effect of the quality of life [10, 11].

### Purpose of this study

We sought to determine the epidemiology of LBP and the disability caused by the symptoms, as well as access and utilization of different health-care resources available to the individuals. This study is based on the results of Stage I, Phase III of the Iranian COPCORD study, which evaluated rheumatologic disorders in three cities.

## Methods

Three cities from the urban phase of the Iranian COPCORD were used for this study. Zahedan, which is situated in south-eastern Iran, with an estimated population of 681,460. Residents of Zahedan are mainly from the Balouch or Fars ethnicities, both considered Caucasian. This city is an underdeveloped region in Iran, and the socioeconomic status is very low [12]. The second city was Sanandaj, located in northwestern Iran. The population is 311,446. Kurds, a subgroup of Caucasians, are the majority in this city. This study was the first study of its kind in the Kurdish population [13]. The third city, Yazd is in central Iran, with a population of 529,673. While the data has not been published yet, this study was conducted only on Zoroastrian people of Yazd, which are of pure Caucasian descent, never having been mixed with other Caucasian subgroups or other ethnicities. Including three cities, from different parts of the country, with vastly different socioeconomic status, and different levels of access to health-care resources, was to provide a clear, undistorted picture of the status quo pertaining to health-care utilization in Iran.

In general, the Iranian health system consists of a universal health-care system which relies on a hierarchical network starting from rural medical centers to urban, tertiary referral centers. While only medical doctors (general practitioners) are licensed to practice medicine, allied health professionals (AHPs), including nurse practitioners, midwives, and physical therapists can visit and treat patients under the supervision of a medical doctor. Complementary and alternative medicine generally consists of unlicensed individuals treating patients with herbal and unconventional therapies.

The sampling plan for all three cities were similar and consisted of cluster sampling based on the population of neighborhoods in the city. Districts and neighborhoods were extracted from the national postal service zip code bank. All interviews were performed by trained physicians or nurse practitioners. The interviewers were local to each city to know the local culture and inhabitants. Interviews were conducted on Fridays (the weekend in Iran) each week on a door-to-door manner, to ensure the presence of as many household members as available. The interviewing team went back to the same cluster on 3 consecutive Fridays to collect data from those who were absent the previous Friday. Every interviewer started data collection by introducing him/herself and showing an identification card, and then continued to fill in the CCQ. After completing the CCQs for each family, questionnaires were submitted to the team head. The head would check and confirm the CCQ.

Data collection for each city was independent, and due to the limited resources, had to be performed sequentially. Therefore, the complete dataset was only ready when the last city was surveyed. Data from Zahedan was surveyed from October 2008 to September 2009. Sanandaj COPCORD data collection was performed between July 2011 and June 2012, and Yazd study was conducted Between July 2014 and July 2015. All data from the three cities were combined after the completion of data collection, and eligible individuals were then queried within this database.

The Farsi translation of the COPCORD Core Questionnaire (CCQ) was used to screen subjects for low back pain. The Farsi version of the CCQ has been previously validated and has been shown to be a reliable and reproducible translation [8, 14]. The CCQ has seven main sections: background information (A), work history (B), Pain/tenderness/swelling/stiffness during the last week (C1) and the past (C2), functional disability (D), difficulty in performing specific tasks (E), treatment (F), and evaluation (G).

In this study, we included individuals who answered positive to any questions in the C2j and C9j sections, which asks about pain/stiffness/tenderness in their back during the last week or the past. Section E, which is the modified Stanford Health Assessment Questionnaire – Disability Index (HAQ-DI), was used to measure the degree of disability due to LBP. Generally, HAQ-DI scores of 0 to 1 are considered no to mild disability, 1 to 2 as moderate, and > 2 as severe disability. Health-care access and utilization are surveyed in sections G2a and G2b. A positive answer to any of the questions in the G2a subsection was considered the utilization of conventional medicine (CM) care. A positive answer to G2b1 (physical therapist) or G2b7 (chiropractor) was considered the utilization of an allied health professional (AHP). A positive answer to other questions in the subsection G2b was considered utilization of complementary and alternative medicine (CAM) providers. Demographic data, including age, gender, ethnicity, and marital status, were collected for each patient. Also, the level of education of each patient was recorded.

### Data analysis

Statistical analysis was performed using IBM SPSS Statistics for Windows, version 24 (IBM Corp, Armonk, NY). Descriptive data were analyzed by survey data analysis methods. Student’s *t-*tests were used to test for differences between groups. The significance level was considered alpha < 0.05 for all tests.

## Results

In total, 9101 patients were interviewed and completed the CCQ in the three cities of Zahedan (2100 individuals), Sanandaj (5001), and Yazd (2000). The prevalence of LBP was 38.6% among all participants (3513 individuals). Sanandaj had the highest prevalence (55.3% of participants), followed by Zahedan (38.8%), and Yazd (17.1%). In total, 71.7% were female and 28.3% were male. The educational level of the participant was as follows: 33.4% no high-school education, 34% some high-school, 19.2% high-school diploma, and 13% had higher education. In patients with LBP, 13.2% were single, 78.5% married, and 8.2% divorced/widowed.

Only 3.3% of patients had not sought any form of care for their LBP, with 66.7% of patients referring to CM providers, 20.8% to AHP, and 9.2% CAM care utilization.

Table 1 summarizes the demographics of LBP patients utilizing professional care, broken down by the type of health-care provider. CM care utilization was significantly higher in female patients (*P* = 0.03). This association was not observed with AHP (*P* = 0.443) or CAM (*P* = 0.718) utilization.

**Table 1:** Demographics of COPCORD patients utilizing health care for back pain

	CM	AHP	CAM
**Mean age (SD)**	47.3 (15.91)	49.9 (14.95)	47.1 (16.03)
**Gender**			
Male, *n*	448	144	69
*%*	*26.4*	*27*	*29.4*
Female, *n*	1247	389	166
*%*	*73.6*	*73*	*70.6*
*Subtotal*	*1695*	*533*	*235*
**Ethnicity**			
Fars	193	*51*	39
*%*	*11.4*	*9.6*	*16.7*
Kurd	1069	*315*	83
*%*	*63.1*	*59.1*	*35.5*
Balouch	145	*38*	38
*%*	*8.6*	*7.1*	*16.2*
Zoroastrian	274	*126*	69
*%*	*16.2*	*23.6*	*29.5*
Other	13	*3*	5
*%*	*0.7*	*0.6*	*2.1*
*Subtotal*	*1694*	*533*	*234*
**Marital status**			
Single	174	44	29
*%*	*10.3*	*8.3*	*12.4*
Married	1353	433	181
*%*	*80*	*81.4*	*77.3*
Divorced/widowed	164	55	24
*%*	*9.7*	*10.3*	*10.3*
*Subtotal*	*1691*	*532*	*234*

The discrepancy in the subtotal rows is due to patients refusing to answer certain questions. *CM* Conventional medicine; *AHP* Allied health professional, *CAM* Complementary and alternative medicine

Also, older age was significantly correlated with CM, AHP, or CAM care utilization (*P* < 0.001). Moreover, using CM (*P* = 0.03) and AHP (*P* = 0.004) care was significantly higher in patients with a high-school diploma, but using CAM care and education level did not have a significant association (*P* = 0.092).

CM, AHP, and CAM care utilization was significantly higher in patients with Zoroastrian ethnicity compared to all other ethnicities (all *P* values < 0.001). CM and AHP care utilization were significantly higher among divorced/widowed patients (*P* values < 0.001). There was no association between CAM care utilization and being divorced/widowed (*P* = 0.468).

Table 2 summarizes the disability severity and education level of individuals utilizing health care for LBP. Patients with higher levels of disability had a significantly higher usage of CM care (*P* < 0.001) and AHP care (*P* < 0.001), but not CAM (*P* = 0.21). Also, patients with a high-school diploma were significantly more likely to utilize CM (*P* = 0.003) and AHP care (*P* = 0.004), but not CAM (*P* = 0.092).

**Table 2:** Disability severity (based on the Health Assessment Questionnaire – Disability Index) and education level of COPCORD patients utilizing health care for back pain

	CM	AHP	CAM
**Disability**			
Mild	226	57	51
*%*	*15.7*	*12.2*	*23*
Moderate	197	59	26
*%*	*13.7*	*12.6*	*11.7*
Severe	1012	351	145
*%*	*70.6*	*75.2*	*65.3*
*Subtotal*	*1695*	*533*	*235*
**Education**			
No high school	251	*149*	64
*%*	*28.9*	*28*	*27.5*
Some high school	320	*190*	81
*%*	*36.9*	*35.6*	*34.8*
High school diploma	169	*126*	57
*%*	*19.5*	*23.6*	*24.4*
College or higher	128	*68*	31
*%*	*14.7*	*12.8*	*13.3*
*Subtotal*	*868*	*533*	*233*

*CM* Conventional medicine, AHP Allied health professionals, *CAM* Complementary and alternative medicine

## Discussion

While access is an indicator of the quality of a health-care system, utilization of the provided health-care depends on various socio-economic and cultural factors, in addition to the health system itself. Knowledge about the patterns of health-care utilization is vital in developing and modifying health-care policies, especially in low- and middle-income countries. To our knowledge, this is the first study to describe the epidemiology of LBP and the utilization of health-care in the Iranian population. Also, differences in characteristics of patients utilizing a variety of health-care resources were studies for the first time in three cities among individuals with a range of socioeconomic status, educational level, access to health-care, and ethnicities.

LBP, as one of the most common musculoskeletal complaints, affects all ages and groups. The chronicity, pain and disability range from a days-long mild pain to a life-long, debilitating pain that leads to early retirement from work, with a multitude of physical, emotional, and psychological disorders that may ensue [7]. The prevalence of individuals with LBP is projected to grow with the increase in life expectancy [15]. In the low- and middle-income countries of the developing world, this may pose a threat to sustained economic growth. Forced early retirement due to disability caused by chronic LBP may hinder the growth of an aging society and deprive it of the limited, vital workforce at hand [16, 17]. In the past two decades, we have witnessed a revolution in the diagnosis and treatment of many rheumatologic disorders, as well as treatment of complications, which has provided millions of patients with a normal or near-normal life, who would otherwise have been suffering from severe disability [18]. The same, unfortunately, cannot be argued about low back pain. Except for patients with LBP secondary to other rheumatologic disorders, non-specific LBP has not seen the significant progress that rheumatologic/immunologic disorders have witnessed [2]. The aforementioned are the main reasons LBP was chosen as the disease-of-interest in this COPCORD sub-study.

Our results show a mean prevalence of 38.6% for LBP among the cities surveyed. This rate is one of the highest reported in the COPCORD studies. The only study with similar results is the Chinese study, performed in Beijing, which showed a 35% prevalence of LBP [19]. More recent COPCORD studies from the Latin American countries indicate a 10.4% prevalence in the Portuguese population and 8% in the Mexican population [10, 11]. While some difference is expected between regions, this stark discrepancy might be rooted in the cultural beliefs and preconceptions regarding medical care. If the individual believes that they would receive a more appropriate care with a more severe complaint, even the most minor LBP is reported in detail.

Several demographic, socioeconomic, medical, and cultural factors have been associated with LBP. Medical comorbidities, unhealthy habits, low educational level, living in rural areas, obesity, other musculoskeletal complaint/disorders, female gender, and anxiety have been highly associated with LBP in different studies [9–11, 19]. While LBP has been found to be a prevalent disease in almost all populations, the burden of the disease is disproportionately carried by the underprivileged populations of the society, with ethnicity, rural residence, inadequate access to healthcare services, and lower educational level being the risk factors for a greater impact of LBP on the patient’s vulnerability for being impacted by the disease [9]. It is noteworthy that while we did not study the LBP associated risk factors and the impact the symptoms have on the patient’s life, these are contributing factors to the burden of LBP in the society and warrant deeper exploration in the future studies.

We found a high rate of utilization of health-care resources among COPCORD interviewees with LBP, with the vast majority (96.7%) of patients utilizing care. Iran has a universal health-care system with a vast network of public-sector health-care resources, which mitigates the financial impediments to health-care access. While we were unable to find studies reporting LBP-specific health-care utilization behavior, but this rate is in the range of middle- to high-income countries with a universal health-care system [20–22]. The high rate of CM utilization is also in the range of more-developed countries.

Female individuals in this study were more likely to utilize CM care, but not AHP or CAM, which is in line with some studies, which have reported the association between female gender and utilization of health-care [23, 24]. However, there are contradictory reports of the association between female gender and CAM care utilization, with some reporting a significant association [20, 25], and some showing no association [20, 25].

The association between ethnicity and health-care utilization has been demonstrated before. Being of an indigenous ethnicity was a risk factor for a lower rate of health-care access and utilization, as well as a higher burden of symptoms of the quality of life [9, 26]. In this study, we found that the Zoroastrian population had the highest rate of health-care utilization of every kind, which might also be affected by their socioeconomic and education levels.

Higher education and higher levels of disability were correlated with utilization of health-care resources in this study. These findings have been reported consistently in previous studies, which shows a tendency for higher educated patients to utilize care irrespective of financial status [20, 24, 27].

Older age was also correlated with utilization of CM and AHP care in this study. Previous studies have also reported similar results in countries with a universal health-care system [27]. This may be due to the elderly being cognizant of their health status or the severity of the symptoms, which was not assessed in this study. Also, older people who visit a health-care provider for another comorbidity in a regular fashion will theoretically utilize the same resources for a new symptom.

This study has some limitations. First, our study population may not be representative of the whole Iranian population. We tried to mitigate this by including data from three cities with different ethnic, cultural, and socioeconomic situations. Second, some individuals refused to answer some questions, especially about ethnicity and educational level. Also, we had to exclude disability index data for some individuals, due to incomplete forms, which reduces the power of this study. We have provided the subtotal number of complete data in the tables. Third, we had no way of quantifying the use of health-care resources, specifically whether the patient was satisfied with the provided care and how likely they are to utilize care again. Finally, due to the heterogeneity in data collection pertaining to the individual’s job and activity level, we did not include them in the analysis, which are potentially influential factors in the prevalence of LBP. This study also has some strengths. The COPCORD study is a well-established program, with standardized evaluations which produces results that are comparable globally. Additionally, the high number of individuals in this study, with an array of socioeconomic and cultural background also adds to the power of this study.

## Conclusions

In conclusion, this study provides meaningful insight on the current pattern of healthcare utilization behavior of the Iranian population, and the factors associated with the utilization of health-care resources in patients with low back pain. The results of this study would be a valuable addition to the literature used in developing health-care programs to combat the increasing incidence of low back pain in the developing world, in hopes of decreasing the pain and disability associated with the disease.

## Data Availability

The datasets used and/or analyzed during the current study are available from the corresponding author on reasonable request. All data analyzed is part of the WHO COPCORD studies.

## Abbreviations

WHO-ILAR: World Health Organization-International League of Associations for Rheumatology
COPCORD: Community Oriented Program for Control of Rheumatic Diseases
LBP: Low-back pain
CM: Conventional medicine
AHP: Allied health provider
CAM: Complementary and alternative medicine
CCQ: COCPORD Core Questionnaire
HAQ-DI: Health Assessment Questionnaire – Disability Index
